# Predictors of fatigue progression in long COVID among young people

**DOI:** 10.1016/j.bbih.2025.100982

**Published:** 2025-03-24

**Authors:** Elias Myrstad Brodwall, Joel Selvakumar, Lise Beier Havdal, Silke Sommen, Lise Lund Berven, Erin Cvejic, Vegard Bruun Bratholm Wyller, Maria Pedersen

**Affiliations:** a Dept. of Pediatrics and Adolescent Health, Akershus University Hospital, Lørenskog, Norway; b Institute of Clinical Medicine, University of Oslo, Oslo, Norway; c Dept. of Clinical Molecular Biology (EpiGen), University of Oslo and Akershus University Hospital, Lørenskog, Norway; d The University of Sydney, School of Public Health, Faculty of Medicine and Health, The University of Sydney, Australia; e Dept. of Pediatric Neurology, Oslo University Hospital, Rikshospitalet, Oslo, Norway

**Keywords:** Post-infective fatigue syndrome (PIFS), Post-COVID condition (PCC), Long COVID, Chronic fatigue, Adolescents, SARS-CoV-2, Fatigue trajectory, Fatigue risk factors

## Abstract

Long COVID, or post-COVID-19 condition (PCC), has emerged as a significant health concern, with fatigue being the most prevalent persistent symptom among young people. However, research on predictors of fatigue progression in young populations is limited. This study aimed to investigate factors during acute SARS-CoV-2 infection that could predict fatigue progression between six and 12 months post-infection in a cohort of young people with chronic fatigue following COVID-19. Data from the Long-Term Effects of COVID-19 in Adolescents (LoTECA) project were analyzed. A total of 93 participants (mean age 18.5 years, 84 % female) with chronic fatigue at six months, completed the 12-month follow-up. Multivariate analyses identified non-European ethnicity, higher interferon gamma (IFN-γ) levels, and lower RR-interval (higher resting heart rate) during acute infection as significant predictors of fatigue progression from six to 12 months. These three factors explained 21 % of the variance in the fatigue score, highlighting the importance of ethnicity, immune response, and autonomic function in the trajectory of long COVID fatigue. Early identification and targeted interventions, particularly for ethnic minorities and those with specific immune or autonomic markers during acute infection, may be helpful in reducing long-term fatigue. Further research is needed to explore treatment strategies for affected young populations.

## Highlights

• Post-COVID fatigue in young people gradually declines the first year post-infection.
• A range of variables are associated with fatigue progression.
• Non-European ethnicity, immune, and sympathetic activity predict fatigue progression.
• Treatment gives a fatigue reduction.

## 1 Introduction

The post-COVID-19 condition (PCC), often referred to as Long COVID, is defined as any symptom that lasts more than two months after acute infection with severe acute respiratory syndrome coronavirus 2 (SARS-CoV-2), causes functional impairment, and lacks an alternative explanation (Soriano et al., 2022). Over 200 symptoms have been associated with PCC (Soriano et al., 2022). However, in a recent systematic review of young people, only fatigue, altered smell/taste, dyspnea and myalgia are significantly more prevalent in the PCC group (prevalence differences ranging from one to seven percent) when compared to SARS-CoV-2 negative control groups (Behnood et al., 2023). Fatigue is the most common and one of the most debilitating persistent symptoms following coronavirus disease 2019 (COVID-19) (Fernández-de-Las-Peñas et al., 2021; Ceban et al., 2022).

Persistent fatigue is also a well-documented phenomenon following infections caused by a wide range of pathogens (Hickie et al., 2006; Pedersen et al., 2019a; Hanevik et al., 2014). While estimates vary, approximately 10–15 % of individuals adhere to the diagnostic criteria for post-infective fatigue syndrome (PIFS) six months after an infectious event from various pathogens (Hickie et al., 2006; Pedersen et al., 2019a; Hanevik et al., 2014; Katz et al., 2018; Sandler et al., 2021; Selvakumar et al., 2023). The PIFS diagnostic criteria shares several characteristics with the PCC definition but is more stringent (Sandler et al., 2021). Furthermore, the risk factors for developing PCC and PIFS overlap to some extent and include female sex, initial symptom burden, and anxiety level (Lim et al., 2020; Bakken et al., 2014). Studies on fatigue syndromes also report higher rates of chronic fatigue in ethnic minorities (Dinos et al., 2009).

Most persistent post-infectious symptoms tend to decrease over time following an infectious event (Hickie et al., 2006). A population-based longitudinal study reports a nearly 50 % recovery rate from fatigue in PCC within two years (Hartung et al., 2024). Risk factors for non-recovery from baseline more than six months after infection are male sex, older age, and lower levels of formal education, as well as depressive symptoms and/or headache at inclusion. However, research on predictors of fatigue progression in PCC and PIFS is limited, and, to the best of our knowledge, no studies have specifically investigated predictors of fatigue progression during the convalescent phase following acute COVID-19-infection.

Research on long-term symptoms in children and young people remains limited compared to adults (Behnood et al., 2023), despite younger populations potentially facing these consequences for a greater proportion of their lives, impacting disability-adjusted life years (DALY) (Nurchis et al., 2020). The implications for public health should be of concern to governments and public health policymakers. Identifying predictors for long-term morbidity is a critical step in designing targeted interventions and effective public health planning.

Hence, the aim of the present study was to investigate the trajectory of fatigue following COVID-19 and to identify biological, psychological, and social factors present during acute SARS-CoV-2 infection that could predict fatigue progression levels from six to 12 months in a cohort of young people with chronic fatigue six months following COVID-19.

## 2 Method

### 2.1 Study design

This study is part of the Long-Term Effects of COVID-19 in Adolescents (LoTECA) project. LoTECA is a prospective observational cohort study of adolescents and young adults between the ages of 12 and 25 with and without acute SARS-CoV-2 infection (ClinicalTrials ID: NCT04686734). Details of the study design are reported elsewhere (Selvakumar et al., 2023). This paper presents baseline predictors for fatigue progression between the six- and 12-month follow-up visits among the participants who were SARS-CoV-2 positive at inclusion and suffered from persistent and clinically relevant fatigue at six months follow-up, defined as a total sum score of four or more on the Chalder Fatigue Questionnaire (CFQ) (bimodal scoring of single items) (White et al., 2011; Jackson, 2015).

### 2.2 Participants

Inclusion period spanned from December 24, 2020 to May 18th^,^ 2021. During this time, individuals between ages 12 to 25 who underwent testing for SARS-CoV-2 via reverse-transcription polymerase chain reaction (RT-PCR) from upper respiratory tract swabs analyzed at either Fürst Medical Laboratories or the Department of Microbiology and Infection Control, Akershus University Hospital, were contacted for participation in this study. Exclusion criteria were: (a) more than 28 days since onset of symptoms or SARS-CoV-2 test, (b) hospitalization due to COVID-19, and (c) pregnancy. Baseline assessment followed a mandatory 10-day quarantine among the SARS-CoV-2 positive participants. All participants, as well as legal guardians where applicable, provided written informed consent. The LoTECA project is approved by the Regional Committee for Medical Research Ethics. Further details on recruitment and inclusion have been described elsewhere (Selvakumar et al., 2023).

### 2.3 Investigational program

At study inclusion and follow-up sessions, participants were summoned to the study center at Akershus University Hospital, Norway. Each standardized session lasted about 2 h. The investigational program included clinical examination, functional tests, blood samples and questionnaires (Selvakumar et al., 2023).

### 2.4 Clinical interview and examination

Participants were asked about chronic diseases, medications, and both their own and their parents’ country of origin. The clinical examination included vital signs (blood pressure, heart rate, respiratory frequency, blood oxygen saturation (SpO_2_), tympanic temperature), height and weight measurements, a standardized clinical review of organ systems, and urine dipstick analysis plus pregnancy test if indicated.

### 2.5 Functional tests

Forced vital capacity (FVC) and forced expiratory volume in 1 s (FEV1) were measured through spirometry (EasyOne® Air spirometer, EasyOne Connect software, NDD Medizintechnic AG, Switzerland), and the FEV1/FVC ratio was calculated.

A 5-min supine resting ECG recording was performed utilizing Bittium Faro 360® (Bittium Corporation, Oulu, Finland). Heart rate variability (HRV) indices were calculated in the time and frequency domains based upon time series of the RR-interval (RRI) according to international standards (Heart rate variability, 1996). Computed time domain indices include SDNN (the standard deviation of all RRIs), pNN50 (the proportion of successive RRIs with a difference greater than 50 ms), and r-MSSD (the square root of the mean square differences of successive RRIs). In the frequency domain, power densities were computed in the low-frequency (LF) band (0.04–0.15 Hz) and the high-frequency (HF) band (0.15–0.5 Hz), and expressed in absolute (LF_abs,_ HF_abs_) units. In addition, the LF_abs_/HF_abs_ ratio was computed. Vagal (parasympathetic) activity is considered the main contributor to HF-variability of heart rate as well as the pNN50 and r-MSSD indices, whereas both vagal and sympathetic activity contributes to LF-variability and the SDNN index (Heart rate variability, 1996).

Tests for cognitive function included the digit span test of working memory, as well as tests of recall, recognition, and verbal learning through Hopkins Verbal Learning Test – Revised (HVLT-R) (Benedict et al., 1998; Grizzle et al., 2011).

### 2.6 Blood samples and laboratory assays

A total of 23 immunological markers in plasma were assayed using multiplex technology and enzyme immunoassays, as described in prior publications (Lund Berven et al., 2022). These included numerous interleukins and interferons, such as interferon gamma (IFN-γ). Specific antibodies (ABs) against both SARS-CoV-2 and Epstein-Barr virus (EBV) were analyzed in serum.

Neurofilament light chain (NfL) and glial fibrillary acidic protein (GFAp) were assayed in serum and are considered markers of neuronal damage and neuroinflammation, respectively (Havdal et al., 2022). Hematological and biochemical analyses including D-dimer, Ferritin, Vitamin B_12_, and cardiac markers troponin T and N-terminal prohormone of Brain Natriuretic Peptide (NT-proBNP), were performed at the accredited laboratory at Akershus University Hospital. Biomarkers were selected based on established hypotheses or prior studies indicating their potential involvement PIFS or PCC.

### 2.7 Questionnaires

A comprehensive questionnaire covered chronic diseases, family history of chronic conditions, nicotine use, substance use, physical activity, parental occupations, vaccination status, and history of COVID-19 (Selvakumar et al., 2023; Lund Berven et al., 2022; Havdal et al., 2022). Parents' occupations were used as a proxy for socio-economic background, in accordance with the International Socio-Economic Index (ISEI) of occupational status (Ganzeboom et al., 1992). Symptoms of fatigue, post-exertional malaise (PEM), sleep quality, pain, anxiety, and depression were chartered through validated inventories, including Chalder Fatigue Questionnaire (CFQ), DePaul Symptom Questionnaire, Karolinska Sleep Questionnaire (KSQ), Brief Pain Inventory (BPI), and Hospital Anxiety and Depression Scale (HADS) (Jackson, 2015; Jason and Sunnquist, 2018; Klepstad et al., 2002; Nordin et al., 2013; Zigmond and Snaith, 1983). Additionally, a questionnaire specifically designed for chronic fatigue research was applied, with minor adjustments specific for COVID-19 (Elliott et al., 2021). Psychological traits, loneliness, significant life events, and quality of life were assessed using validated inventories, including the Penn State Worry Questionnaire (PSWQ), UCLA Loneliness Scale, Life Event Checklist (LEC), and Pediatric Quality of Life Inventory (PedsQL), among others (Selvakumar et al., 2023; Pallesen et al., 2006; Russell et al., 1980; Gray et al., 2004; Varni et al., 2003).

In this study, ethnicity was categorized dichotomously as European or non-European, with participants classified as non-European if at least one parent originated from outside Europe. Non-European countries represented in this group included Afghanistan, unspecified regions of Asia, Colombia, China, Ghana, India, Iraq, Morocco, Pakistan, Philippines, Somalia, South Korea, Sri Lanka, Sudan, Turkey, and Vietnam. Origin from Chechnya was also included in the non-European group.

### 2.8 Register linkage

Vaccination status of participants against SARS-CoV-2, including dosage, dates of administration, and manufacturers, was obtained through linkage with the SYSVAK register administered by the Norwegian Institute of Public Health (Nasjonalt vaksinasjonsregister SYSVAK - Norwegian Immunisation Registry SYSVAK).

### 2.9 Statistical analyses

Variables treated as continuous are presented as mean ± standard deviation (SD) for normally distributed data or median with interquartile range (IQR) for non-normally distributed data. Categorical variables are reported as counts and percentages. Differences in fatigue scores (Chalder Fatigue Questionnaire, CFQ, using the continuous Likert scoring) between the six- and 12-month follow-ups were analyzed using paired-sample t-tests. For prediction analyses of fatigue progression, the 12-month CFQ score was applied as the dependent variable, controlling for the six-month CFQ value.

More than 175 initial variables were categorized into eight groups: background/constitutional, organ affection/clinical findings, immunological markers, autonomic markers, cognitive function tests, clinical symptoms, psychological traits/emotions, and functional capabilities/life events (eSupplementary Table 2). Linear regression was performed for each potential predictor variable against the dependent variable. Predictor variables with an association of p < .1–12 months fatigue score (controlled for six-months fatigue score) were subsequently included in backward multiple regression analyses within each category. An initial p-value threshold of <0.1 was selected to avoid premature exclusion of potentially relevant variables. A more stringent p-value of <0.05 was applied in the final models to ensure robustness. The variables with a remaining p < .05 within each category were included in a final multiple regression analysis, combining variables from all categories into a final model. As incomplete data sets were confined to 5 cases (5,4 %), the missing data was considered negligible, and no imputation was performed. Pearson correlation was conducted to evaluate the intercorrelation between the predictor variables included in the multiple regression analysis.

Participants who participated at six months follow-up but declined the 12-month follow-up anecdotally reported recovery as the primary reason for dropping out. Attrition analyses were conducted to compare the characteristics of participants who dropped out with those who completed the 12-month follow-up.

All statistical analyses were performed with SPSS version 29.0 (SPSS Inc, Chicago, IL).

## 3 Results

**Fig. 1:**
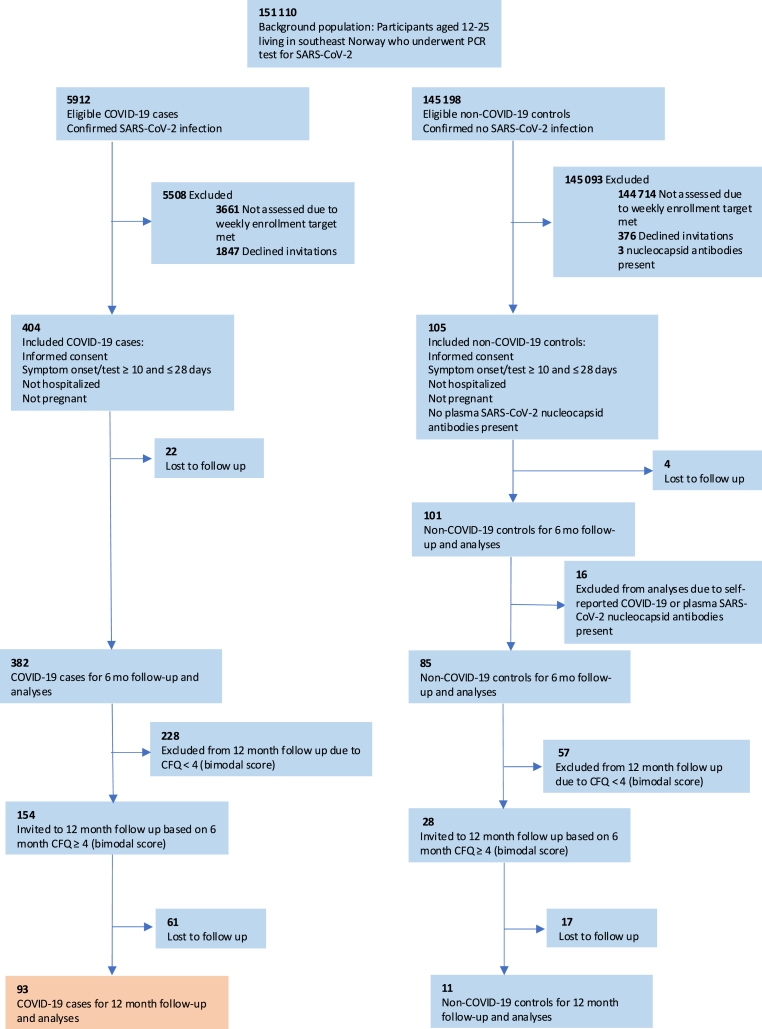
Flowchart of LoTECA.

A total of 404 SARS-CoV-2 positive individuals were included in the study. Of these, 382 completed the six-month follow-up, of whom 154 (40 %) had a CFQ score of four or more (bimodal scoring of single items), making them eligible for the 12-month follow-up (Fig. 1).

A total of 93 participants (84 % females, mean age 18.5 years at baseline) completed the 12-month follow-up assessment (Table 1).

**Table 1:** Baseline characteristics.

		**N**
Sex - number of females (%)	78 (83.9%)	93
Age at baseline, years - mean (SD)	18.5 (4.2)	93
Ethnicity - number of Europeans (%)	66 (71.0%)	93
Number of days since onset of symptoms - mean (SD)	18.5 (4.2)	93
BMI, kg/m^2^ - mean (SD)	23.3 (4.3)	93
Vaccinated at baseline - number (%)	2 (2.2%)	93
… 0 doses	91 (97.8%)	
… 1 dose	2 (2.2%)	
… 2 doses	0 (0%)	
Comorbidity (incl. asthma) - number (%)	27 (30.0%)	90∗
Highest ISEI-08^a^ rank of parent - mean (SD)	58.6 (20.8)	84∗
Family member with chronic disease^b^ - number (%)	42 (46.7%)	90∗
Chalder Fatigue Questionnaire^c^ baseline, total score - mean (SD)	20.0 (4.9)	90∗
Chalder Fatigue Questionnaire 6 mo, total score - mean (SD)	19.8 (4.4)	93
Chalder Fatigue Questionnaire 12 mo, total score - mean (SD)	18.8 (5.2)	93

^a^ The International Socio-Economic Index of Occupational Status (ISEI) combines income and education to reflect the status of an occupation, higher score implies higher socioeconomic status.

^b^ Having a sibling or parent affected by chronic disease, self-reported.

^c^ Chalder Fatigue Questionnaire (CFQ); 11 items scored on 4-point Likert scales, sum score 0–33, higher score implies more fatigue.

∗ At baseline, three participants did not complete the questionnaire, resulting in missing questionnaire data including CFQ for these individuals. For ISEI, the number of missing was nine.

**Table 2:** Final multiple regression model with CFQ 12 months as dependent variable.

Baseline independent variable	Linear regression coefficient B (CI)	p-value	Δadj.R^2^
Level of fatigue at six months^a^	0.555 (0.369–0.741)	<0.001	0.238
Ethnicity^b^	3.769 (1.862–5.677)	<0.001	0.103
RR-interval, ms^c^	−0.008 (−0.016 to −0.001)	0.036	0.044
IFN-γ, pg/mL^d^	0.244 (0.064–0.423)	0.008	0.048
Explained variance (adj.R^2^) of model^e^			0.446

^a^ Fatigue was measured by Chalder Fatigue Questionnaire (CFQ), total score 0 to 33; higher score implies more fatigue.

^b^ Ethnicity was classified as either European (scored as 0) or non-European (scored as 1) based on country of birth of participant and participant's parents.

^c^ Mean of all normal RR-intervals during 5-min resting ECG.

^d^ Plasma interferon (IFN)-γ, pg/mL.

^e^ The explained variance without the level of fatigue at six months was 0.208.

The 61 individuals who declined the 12-month follow-up assessments were broadly similar to those who attended, including comparable CFQ scores at baseline and six months. However, they reported a higher quality of life and had a lower resting heart rate (higher RR-interval) (eSupplementary Table 1).

There was a non-significant trend toward a lower CFQ score from six months to 12 months (p = .058), with a mean reduction in fatigue score of 0.97 (SD 4.87), which followed a normal distribution. There was a significant reduction in fatigue score (CFQ) from baseline to 12 months (p = .043), with a mean reduction in fatigue score of 1.3 (SD 6.0).

Bivariate linear regression analyses identified more than 40 baseline variables with a p-value <0.1 associated with CFQ total score at 12 months when adjusted for the CFQ total score at six months (eSupplementary Table 2). These included constitutional, immunological, autonomic, and psychological variables. Correlation analysis revealed strong intercorrelations within the same categories (eSupplementary Figure 1).

Multivariate modeling identified three independent predictors significantly associated with an increase in fatigue score between six and 12 months: non-European ethnicity (3.77 [1.86 to 5.68], p < .001), interferon gamma (IFN-γ) (0.24 [0.06 to 0.42], p = .008), and RR-interval (RRI) (−0.008 [−0.016 to −0.001], p = .036) (Table 2).

Hence, fatigue progression was significantly associated with non-European ethnicity, higher levels of IFN-γ, and lower RRI (indicating higher resting heart rate). This model explained 45 % of the variance in fatigue development, with an adjusted R^2^ of 0.21 after accounting for the variance attributed to the CFQ score at six months.

In the non-European ethnicity group, 74 % had background from Asia and 22 % from Africa (eSupplementary Table 3). Non-European ethnicity correlated with several variables that were associated with fatigue progression in bivariate analyses, including higher IgG and glucose levels, a higher resting heart rate, lower levels of physical activity and lower self-efficacy (eSupplementary Figure 1).

At baseline, the non-European (M = 19.6, SD = 6.3) and European (M = 20.2, SD = 4.3) groups had comparable levels of fatigue (CFQ sum score), p = .656. Fatigue sum scores at six months were not significantly different between the European (M = 19.7, SD = 4.6) and non-European (M = 20.2, SD = 4.1) groups, p = .627. The CFQ sum scores at 12 months, however, were significantly different between the groups, with more fatigue in the non-European (M = 22.0, SD = 4.5) versus the European (M = 17.5, SD = 4.9) group, p < .001.

Relationships between ethnicity and psychosocial questionnaire scores were examined to enhance interpretability of the ethnicity component. Ethnicity did not show a significant correlation with the Brief Illness Perception Questionnaire (BIPQ) (r(88) = 0.11, p = .33), the Body Vigilance Scale (BVS) (r(88) = 0.13, p = .23), or other measures, except for the depression subscale of the Hospital Anxiety and Depression Scale (HADS D) (r(88) = 0.25, p = .016) and the General Self-Efficacy Scale short form (GSE-6) (r(88) = −0.30, p = .004). In the GSE-6, Europeans (M = 17.4, SD = 2.3) compared to non-Europeans (M = 15.3, SD = 4.2) reported significantly higher scores, t(88) = 2.4, p = .025. In the HADS D, non-Europeans (M = 7.9, SD = 4.0) report more depression than Europeans (M = 5.7, SD = 3.7), t(88) = −2.5, p = .016.

Fatigue progression between six and 12 months showed no significant association with adherence to the WHO's diagnostic criteria for PCC at six months (p = .293), but there was a marginal association with PIFS as defined by the Fukuda criteria at six months (p = .094) (eSupplementary Tab. 2). Patients who reported treatment between six and 12 months tended towards less fatigue progression (p = .072) (eSupplementary Table 2 and eSupplementary Table 4).

Few biomarkers from the routine laboratory analyses displayed any significant association with fatigue progression (eSupplementary Table 2), and none were included in the final model.

## 4 Discussion

The main results from this study indicate a trend toward reduced fatigue levels from six to 12 months after COVID-19 infection, and a significant reduction in fatigue from baseline to 12 months. The most significant predictors for fatigue progression, i.e. more fatigue, were non-European ethnicity, higher baseline INF-γ levels, and lower baseline RRI (i.e. a higher resting heart rate).

The marginally non-significant reduction in fatigue observed between six and 12 months may be explained by the fact that participants who experienced improvements seemed less likely to attend the 12-month follow-up. This could result in a biased sample at the 12-month time point, potentially obscuring further improvements in fatigue.

In the present study, non-European ethnic origin emerged as the strongest predictor of fatigue progression during the observation period. Ethnic minority status has been identified as a significant risk factor for both severe acute COVID-19 and the development of long-term symptoms (Subramanian et al., 2022). Potential explanations include genetic susceptibility, disparities in access to health care and personalized information, socioeconomic level, and cultural background (Tai et al., 2021). However, several studies also report contrasting findings on ethnicity (Khunti et al., 2024), suggesting that non-genetic factors might offer more meaningful explanations.

Interestingly, though non-European ethnicity correlated with lower socioeconomic ranking in our study, the latter was not associated with fatigue progression. Furthermore, ethnicity was associated with several immunological, autonomic, lifestyle and social risk factors for fatigue progression, reflecting the complexity of this variable (eSupplementary Figure 1).

Illness beliefs, influenced by accurate information from health care providers, are critical according to several studies (Burton et al., 2024; Rozenkrantz et al., 2022). If ethnicity influences access to accurate healthcare information and if illness beliefs vary across cultures, these factors may mediate the observed effects of ethnicity on health outcomes. Yet, ethnicity did not correlate with (Klepstad et al., 2002) on illness belief in our data, but rather on self-efficacy (GSE-6). Higher scores on the self-efficacy questionnaire are indicative of more social support, better mental health, and active problem-focused coping (Romppel et al., 2013). Scores on self-efficacy in the non-European group did not seem to correlate with fatigue scores however, though our sample size is too small to conclude. Additionally, the non-European group scored higher on depression, a known factor associated with fatigue (Selvakumar et al., 2023; Pedersen et al., 2019b).

Certain symptoms, like pain and fatigue, are reported more frequently by some ethnic groups, and this is partly culture-dependent (Dinos et al., 2009; Palmer et al., 2007). This is controlled for in this study by including the fatigue level at six months in the modeling. While fatigue levels declined with time for the European group, the opposite was true for the non-European individuals. This study was conducted during a time with pandemic measures, and it is possible that these restrictions may have affected the ethnic groups in different ways, contributing to the difference in fatigue levels. This hypothesis is strengthened by the fact that COVID-positivity at baseline did not predict symptoms at six months (Selvakumar et al., 2023). Our sample size did not allow for subgroup analyses of mediation. Therefore, further research is warranted to understand the impact of ethnicity on the progression of fatigue following COVID-19.

High heart rate during acute SARS-CoV-2 infection was also a predictor of fatigue progression from six to 12 months. In the final model, the RR-interval (the inverse of heart rate) remained a significant independent predictor with a negative coefficient. However, other markers, such as pNN50, r-MSSD, SDNN, and LF_abs_, showed significant associations in bivariate analyses, with all autonomic variables demonstrating consistent trends coherent with an increased sympathetic activity and decreased parasympathetic activity (eSupplementary Table 2). Interestingly, baseline symptoms potentially linked to high sympathetic nervous activity, such as orthostatic dizziness and cold and pale hands, were also associated with fatigue progression from six to 12 months. Taken together, these results suggest a positive prognostic effect of parasympathetic over sympathetic heart rate control at rest. Previous studies have demonstrated that patients with PCC (Pan et al., 2021), PIFS (Pedersen et al., 2019b) and CFS (Wyller et al., 2008, 2009) exhibit increased sympathetic activity and reduced parasympathetic activity, which aligns with our findings.

Higher baseline levels of IFN-γ were associated with fatigue progression from six to 12 months. IFN-γ is a cytokine involved in numerous immune processes, including responses to infections and cancers, and it can also play a role in autoimmune conditions by prolonging and intensifying inflammatory processes (Kak et al., 2018). IFN-γ related fatigue has already been described in other conditions (Pokryszko-Dragan et al., 2012). Previous research has identified immunological markers as risk factors for post-COVID condition (PCC) and post-infectious fatigue syndrome (PIFS) (Pedersen et al., 2019a; Blomberg et al., 2021). The influence of the immune system in these conditions is complex. A modulation of the immune profile has been observed in children following SARS-CoV-2 infection; however, this modulation did not significantly differ between children who fully recovered and those who developed post-COVID fatigue (Buonsenso et al., 2022).

In the bivariate analysis, immunoglobulin G (IgG) was associated with fatigue progression in a manner consistent with our IFN-γ findings (eSupplementary Table 2). However, IgG did not remain in the final model, possibly due to its correlation with heart rate variability measures (eSupplementary Figure 1). Notably, our correlation analysis revealed a significant association between IFN-γ and symptoms such as pale and cold hands, which are indicative of autonomic dysfunction (eSupplementary Figure 1). These findings support previous evidence of an interaction between the autonomic nervous system and the immune system in relation to long-term symptoms following COVID-19 (Pan et al., 2021).

The association between compliance with the WHO's PCC criteria at six months and subsequent change in fatigue was non-significant. In contrast, a diagnosis of PIFS based on the Fukuda definition at the same timepoint showed a trend towards significant correlation with fatigue progression over the following six months. This further calls into question the utility of the WHO's criteria, being too broad and unspecific, as highlighted in previous publications (Selvakumar et al., 2023).

It is important to note that this study examines changes in fatigue among adolescents based on their fatigue levels six months after the acute infection. This is fundamentally different from investigating risk factors for developing fatigue following a viral infection. In this study, participants are already fatigued, and we are focusing on risk factors for the subsequent changes from that point onward.

The results of this study suggest that targeted interventions for ethnic minorities may be beneficial. Furthermore, interventions targeting the balance between sympathetic and parasympathetic activity should be explored. Additionally, it may be advantageous for the treatment to start as early as possible since fulfilling the PIFS diagnosis at six months implies increased risk of fatigue progression. Participants who received treatment for post-COVID symptoms between the six- and 12-month follow-up showed a tendency towards reduced fatigue compared to those who did not, especially the few who specified type of treatment (eSupplementary Tab. 3). These considerations should be further evaluated in treatment studies.

### 4.1 Strengths and limitations

The LoTECA investigational program was designed to ensure meticulous testing of all participants immediately after time of infection and at follow-up appointments. There were minimal missing data.

Approximately 60 % of participants attended the 12-month follow-up, with most dropouts citing recovery as the main reason for not returning, consistent with findings from other recovery studies (Hartung et al., 2024). Attrition analyses revealed that the dropout group reported a higher quality of life and had a lower resting heart rate (longer RR-interval) compared to those who completed the study. This dropout pattern introduces uncertainty regarding how inclusion of these participants might have impacted the results. The 93 participants who completed the study likely represent a biased sample skewed toward those with less improvement. Furthermore, like all studies of similar design, the initial cohort may have been subject to self-selection bias. Though the initial cohort in LoTECA includes a negative control group, too few participants attended the 12-month follow-up for inclusion in these analyses. Lastly, the study was conducted in a predominantly immune-naïve sample during a period when adolescents were likely impacted by the adverse effects of public health measures and other pandemic-related stressors. These factors may have contributed to higher symptom prevalence and intensity in our sample compared to individuals in the current time.

## 5 Conclusion

The fatigue levels in chronic fatigued adolescents and young adults after an acute COVID-19 infection showed a gradual decline the first year after infection.

Main predictors from the early convalescent stage for fatigue progression between six and 12 months were non-European ethnicity, higher levels of IFN-γ and a higher resting heart rate (i.e. a lower RR-interval).

Our findings highlight the importance of early identification of individuals at risk for long-term fatigue and reinforce the need for further research on this condition, including investigations into potential treatment interventions.

## CRediT authorship contribution statement

**Elias Myrstad Brodwall:** Writing – review & editing, Writing – original draft, Investigation, Formal analysis, Data curation, Conceptualization. **Joel Selvakumar:** Writing – review & editing, Investigation, Data curation. **Lise Beier Havdal:** Investigation. **Silke Sommen:** Writing – review & editing. **Lise Lund Berven:** Validation, Resources, Methodology, Investigation. **Erin Cvejic:** Writing – review & editing, Supervision, Formal analysis, Data curation, Conceptualization. **Vegard Bruun Bratholm Wyller:** Writing – original draft, Validation, Supervision, Resources, Project administration, Methodology, Funding acquisition, Formal analysis, Data curation. **Maria Pedersen:** Writing – original draft, Validation, Supervision, Formal analysis, Conceptualization.

## Funding

This work was supported by: The 10.13039/501100005416Norwegian Research Council [grant #302079], and the DAM foundation [grant #2022/F0387180], as well as institutional support from Dept. of Pediatrics and Adolescent Medicine, 10.13039/501100012446Akershus University Hospital, and Institute of Clinical Medicine, 10.13039/501100005366University of Oslo.

## Declaration of competing interest

The authors declare the following financial interests/personal relationships which may be considered as potential competing interests: Elias Brodwall reports financial support was provided by Dam Foundation. Vegard Bruun Bratholm Wyller reports financial support was provided by Research Council of Norway. If there are other authors, they declare that they have no known competing financial interests or personal relationships that could have appeared to influence the work reported in this paper.

## Data Availability

Data will be made available on request.
