# Supplementary material for: Predictors of fatigue progression in long COVID among young people
Source: Brain Behav Immun Health. 2025 Mar 24;45:100982. doi: 10.1016/j.bbih.2025.100982 (PMC11985126; doi:10.1016/j.bbih.2025.100982)
Supplement: Multimedia component 1 [file mmc1.docx]

| eTable1. Comparison in baseline variables between the fatigued participants at 6 months who attended 12 month follow-up and the fatigued participants at 6 months who did not attend 12 month follow-up (dropouts). | | | | |
| --- | --- | --- | --- | --- |
| Variable | **Participants, n = 93** | **Dropouts,**  **n = 61** | **t-test**  **p-value** | **Bonferroni-corrected**  **p-values** |
| Sex - number of females (%) | 78 (83.9%) | 45 (73.8%) | 0.126 | 6.93 |
| Age at baseline, years - mean (SD) | 18.4 (3.5) | 19.0 (3.5) | 0.297 | 16.335 |
| Ethnicity - number of Europeans (%) | 66 (71.0%) | 49 (80.3%) | 0.191 | 10.505 |
| Number of days since onset of symptoms - mean (SD) | 18.5 (4.2) | 19.0 (5.2) | 0.505 | 27.775 |
| BMI, kg/m^2^ - mean (SD) | 23.3 (4.3) | 23.6 (5.1) | 0.744 | 40.92 |
| BMI z-score - mean (SD) | 0.6 (1.1) | 0.6 (1.2) | 0.950 | 52.25 |
| Vaccinated at baseline - number (%) | 2 (2.2%) | 2 (3.3%) | 0.667 | 36.685 |
| ... 0 doses | 91 (97.8%) | 59 (96.7%) |  |  |
| ... 1 dose | 2 (2.2%) | 2 (3.3%) |  |  |
| ... 2 doses | 0 (0%) | 0 (0%) |  |  |
| Weight, kg - mean (SD) | 66.0 (14.2) | 69.4 (16.5) | 0.165 | 9.075 |
| Height, cm - mean (SD) | **167.9 (8.7)** | **171.4 (9.5)** | **0.019** | 1.045 |
| Comorbidity - number (%) | 27 (29.0%) | 13 (21.3%) | 0.302 | 16.61 |
| Asthma - number (%) | **13 (14.0%)** | **2 (3.3%)** | **0.030** | 1.65 |
| Ever smoked a cigarette - number (%) | 28 (30.1%) | 18 (29.5%) | 0.938 | 51.59 |
| Alcohol - Ever had more than 1 glass of beer, wine or liquor - number (%) | 53 (57.0%) | 32 (52.5%) | 0.575 | 31.625 |
| Ever tried a narcotic drug - number (%) | 16 (17.2%) | 12 (19.7%) | 0.696 | 38.28 |
| Highest ISEI-08 rank of parent - mean (SD) | 58.6 (20.8) | 54.5 (20.0) | 0.242 | 13.31 |
| Family member with chronic disease - number (%) | 42 (45.2%) | 37 (60.7%) | 0.055 | 3.025 |
| Resident/home |  |  | 0.131 | 7.205 |
| ... Both biological parents - number (%) | 51 (54.8%) | 34 (55.7%) |  |  |
| ... Biological mom (including a potential stepdad) - number (%) | 16 (17.2%) | 7 (11.5%) |  |  |
| ... Biological dad (including a potential stepmom) - number (%) | 6 (6.5%) | 1 (1.6%) |  |  |
| ... Adoptive parents - number (%) | 0 (0%) | 0 (0%) |  |  |
| ... Institution/foster care - number (%) | 2 (2.2%) | 0 (0%) |  |  |
| ... Alone - number (%) | 6 (6.5%) | 3 (4.9%) |  |  |
| ... Other (including shared accomodation) - number (%) | 9 (9.7%) | 14 (23.0%) |  |  |
| Heart rate, bpm - mean (SD) | 75.0 (12.6) | 70.8 (13.6) | 0.076 | 4.18 |
| ALAT ^a^, U/L - mean (SD) | 19.1 (17.5) | 22.2 (23.3) | 0.371 | 20.405 |
| NTproBNP ^b^ , ng/L - mean (SD) | 45.6 (35.4) | 44.8 (33.7) | 0.887 | 48.785 |
| B-BasoGran, 10^9^/L - mean (SD) | 0.0 (0.02) | 0.0 (0.02) | 0.870 | 47.85 |
| S-Glucose, mmol/L - mean (SD) | 5.1 (0.7) | 5.0 (0.7) | 0.809 | 44.495 |
| SARS-CoV-2 RBD antibodies, all subtypes ^c^ - median (IQR) | 1005.6 (1621.5) | 1044.4 (1450.6) | 0.833 | 45.815 |
| P-IgG ^d^ , g/L- mean (SD) | 11.1 (2.4) | 11.3 (1.8) | 0.599 | 32.945 |
| IFN-γ ^e^ , pg/mL - median (IQR) | 1.1 (1.5) | 1.5 (2.0) | 0.547 | 30.085 |
| pNN50 ^f^ , % - median (IQR) | 38.6 (37.8) | 46.7 (31.2) | 0.189 | 10.395 |
| RR-interval ^g^, ms - mean (SD) | **854.1 (111.7)** | **901.0 (112.5)** | **0.012** | 0.66 |
| LF_abs_ (ln-transformed) ^h^ , ms^2^ - mean (SD) | 6.3 (1.0) | 6.6 (1.5) | 0.229 | 12.595 |
| Tinnitus ^i^, range 1 to 5 - mean (SD) | 2.0 (1.1) | 2.0 (1.2) | 0.906 | 49.83 |
| Hyperacusis, range 1 to 5 - mean (SD) | 2.1 (1.4) | 2.0 (1.3) | 0.488 | 26.84 |
| Dizziness when standing, range 1 to 5 - mean (SD) | 2.7 (1.4) | 2.8 (1.4) | 0.801 | 44.055 |
| Muscle soreness, range 1 to 5 - mean (SD) | 2.3 (1.3) | 2.2 (1.2) | 0.668 | 36.74 |
| Pale and cold hands, range 1 to 5 - mean (SD) | 2.4 (1.4) | 2.2 (1.4) | 0.555 | 30.525 |
| HADS Anxiety index ^j^ - mean (SD) | 8.5 (4.5) | 7.4 (4.5) | 0.147 | 8.085 |
| HADS sum ^k^ - mean (SD) | 14.9 (7.4) | 13.2 (7.3) | 0.182 | 10.01 |
| Penny State Worry Questionnaire (PSWQ) sum ^l^ - mean (SD) | 52.3 (14.0) | 48.6 (15.5) | 0.125 | 6.875 |
| General Self-Efficacy Scale, short form (GSE-6) sum ^m^ - mean (SD) | 16.8 (3.1) | 17.5 (3.4) | 0.159 | 8.745 |
| Pediatric Quality of Life (PedsQL) sum/average ^n^ - mean (SD) | **61.9 (16.0)** | **68.1 (15.2)** | **0.020** | 1.1 |
| PedsQL physical ^o^ - mean (SD) | 56.6 (22.0) | 63.4 (22.4) | 0.070 | 3.85 |
| PedsQL emotional ^p^ - mean (SD) | 54.9 (21.9) | 60.0 (20.0) | 0.151 | 8.305 |
| PedsQL social ^q^ - mean (SD) | 83.7 (18.3) | 88.6 (14.3) | 0.083 | 4.565 |
| PedsQL psychosocial ^r^ - mean (SD) | **64.7 (16.5)** | **70.6 (14.8)** | **0.028** | 1.54 |
| Physical activity, self-reported, compared to peers - mean (SD) | 5.8 (2.0) | 6.3 (2.3) | 0.219 | 12.045 |
| Sum score of all pos life events in past, Life Event Checklist (LEC) - mean (SD) | **0.6 (1.4)** | **0.2 (0.8)** | **0.047** | 2.585 |
| Chalder Fatigue Questionnaire baseline, total score - mean (SD) | 20.0 (4.9) | 19.3 (5.9) | 0.404 | 22.22 |
| Chalder Fatigue Questionnaire 6 mo, total score - mean (SD) | 19.8 (4.4) | 18.9 (4.1) | 0.212 | 11.66 |
| Chalder Fatigue Questionnaire 12 mo, total score - mean (SD) | 18.8 (5.2) | NA | NA |  |
| Change in fatigue between 6 and 12 months | 0.968 (4.9) | NA | NA |  |
|  |  |  |  |  |
| Bonferroni corrected alpha = 0.05/55 = | 0.000909091 |  |  |  |
| Comments:  Variables selected generally display a p < 0.1 in simple regression with the fatigue at 12 month follow-up, corrected for fatigue level at 6 months follow-up.  a) P-ALAT = Plasma alanine aminotranferase  b) P-NTproBNP = Plasma N-terminal pro-brain natriuretic peptide  c) SARS-CoV-2 RBD antibodies, all subtypes = IgG-type antibody directed against the receptor-binding domain (RBD) of the SARS-CoV-2 virus  d) P-IgG = Plasma Immunoglobulin G  e) P-IFN-γ = Plasma interferon gamma  f) pNN50 = the proportion of successive RRIs with a difference greater than 50 ms  g) RR-interval = Mean of all normal RR-intervals during 5-minute resting ECG  h) LF_abs_ (ln-transformed) = average energy density in the low frequency band, transformed for normal distribution  i) Symptom scores: 5-point Likert scale, 3 indicates 1-2 times a week  j) HADS Anxiety = Hospital Anxiety and Depression Scale, anxiety subscale  k) HADS = Hospital Anxiety and Depression Scale  l) PSWQ = Penn State Worry Questionnaire  m) GSE = General Self-Efficacy Scale, short form (GSE-6)  n) PedsQL = Pediatric Quality of Life  o) PedsQL physical = Pediatric Quality of Life physical functioning  p) PedsQL psychosocial = Pediatric Quality of Life psychosocial functioning, based on the emotional, social, and school functioning subscales  q) PedsQL social = Pediatric Quality of Life social functioning  r) PedsQL emotional = Pediatric Quality of Life emotional functioning | | | | |

| eTable 2. Bivariate linear regression between all baseline varables with fatigue level at 12 months as dependent varable controlling for fatigue level at six months. | | |  |
| --- | --- | --- | --- |
|  |  | CFQ 12 months | |
| Background/constitutional |  | B (CI) | p-value |
| Sex - number of females (%) | 78 (83.9%) | -1.948 (-4.454 to 0.558) | 0.126 |
| Age at baseline - mean (SD) | 18.5 (4.2) | 0.017 (-0.252 to 0.285) | 0.902 |
| Ethnicity ^a^ - number of Europeans (%) | 66 (71.0%) | -4.249 (-6.105 to -2.394) | **< 0,001** |
| Number of days since onset of symptoms - mean (SD) | 18.5 (4.2) | 0.165 (-0.056 to 0.385) | 0.142 |
|  |  |  |  |
| Vaccinated at baseline - number (%) | 2 (2.2%) | 2.982 (-3.424 to 9.389) | 0.358 |
| ... Zero doses | 91 (97.8%) |  |  |
| ... One dose | 2 (2.2%) |  |  |
| ... Two doses | 0 (0%) |  |  |
| Body Mass Index (BMI) *, kg/m2 - mean (SD) | 23.3 (4.3) | -0.120 (-0.341 to 0.100) | 0.280 |
| BMI z-score ^b^ - mean (SD) | 0.6 (1.1) | -0.370 (-1.235 to 0.496) | 0.398 |
|  |  |  |  |
| Ever smoked a cigarette - number (%) | 28 (31.1%) | -2.786 (-4.781 to -0.791) | **0.007** |
| Smoking/cigarette frequency - number (%) |  | 1.231 (-0.827 to 3.290) | 0.229 |
| ... Never | 62 (68.9%) |  |  |
| ... Less than once a month | 24 (26.7%) |  |  |
| ... Two to three times a month | 0 (0%) |  |  |
| ... One to two times a week | 3 (3.3%) |  |  |
| ... Three to five times a week | 1 (1.1%) |  |  |
| ... Every day/Almost every day | 0 (0%) |  |  |
|  |  |  |  |
| Alcohol - Ever had more than one glass of beer, wine or liquor - number (%) | 53 (58.9%) | -1.619 (-3.533 to 0.296) | **0.097** |
| Alcohol frequency - number (%) |  | -1.052 (-2.074 to -0.030) | **0.044** |
| ... Never | 37 (41.1%) |  |  |
| ... Less than once a month | 32 (35.5%) |  |  |
| ... Two to three times a month | 15 (16.7%) |  |  |
| ... One to two times a week | 6 (6.7%) |  |  |
|  |  |  |  |
| Ever tried a narcotic drug (like marihuana, ecstasy or amphetamine) - number (%) | 16 (17.2%) | -2.488 (-4.931 to -0.045) | **0.046** |
| Drug frequency - number (%) |  | 4.832 (-1.306 to 10.970) | 0.113 |
| ... Never | 74 (79.6%) |  |  |
| ... Less than once a month | 13 (14.0%) |  |  |
| ... Two to three times a month | 3 (3.2%) |  |  |
| ... Weekly or more frequent | 0 (0%) |  |  |
|  |  |  |  |
| Highest ISEI-08 rank of parent *^c^ - mean (SD) | 58.6 (20.8) | -0.013 (-0.062 to 0.036) | 0.607 |
| Asthma - number (%) | 13 (14.0%) | 2.280 (-0.420 to 4.980) | **0.097** |
| Any chronic disease including asthma - number (%) | 27 (29.0%) | 1.868 (-0.184 to 3.920) | **0.074** |
|  |  |  |  |
| Family member with chronic disease ^d^ - number (%) | 42 (45.2%) | -0.018 (-1.973 to 1.936) | 0.985 |
|  |  |  |  |
| Number of siblings |  | 0.246 (-0.584 to 1.077) | 0.557 |
| ... Zero siblings - number (%) | 11 (11.8%) |  |  |
| ... One sibling - number (%) | 43 (46.2%) |  |  |
| ... Two siblings - number (%) | 24 (25.8%) |  |  |
| ... Three siblings - number (%) | 7 (7.5%) |  |  |
| ... Four siblings - number (%) | 3 (3.2%) |  |  |
| ... Five siblings - number (%) | 1 (1.1%) |  |  |
| ... Six siblings - number (%) | 0 (0%) |  |  |
| ... Seven siblings - number (%) | 1 (1.1%) |  |  |
|  |  |  |  |
| Resident/home |  |  |  |
| ... Both biological parents - number (%) | 51 (54.8%) |  |  |
| ... Biological mom (including a potential stepdad) - number (%) | 16 (17.2%) | 0.582 (-2.074 to 3.238) | 0.664 |
| ... Biological dad (including a potential stepmom) - number (%) | 6 (6.5%) | -1.628 (-5.476 to 2.220) | 0.402 |
| ... Adoptive parents - number (%) | 0 (0%) |  |  |
| ... Institution/foster care - number (%) | 2 (2.2%) | -0.028 (-6.497 to 6.440) | 0.993 |
| ... Alone - number (%) | 6 (6.5%) | 0.625 (-3.208 to 4.458) | 0.746 |
| ... Other (including shared accomodation) - number (%) | 9 (9.7%) | -3.988 (-7.364 to -0.613) | **0.021** |
|  |  |  |  |
|  |  | CFQ 12 months | |
| Organ affection/clinical findings |  | B (CI) | p-value |
| Peripheral blood oxygen saturation (SpO_2_)_,_ % - mean (SD) | 98.5 (1.1) | -0.087 (-0.931 to 0.756) | 0.837 |
| Respiratory rate per minute - mean (SD) | 16.6 (4.2) | 0.149 (-0.076 to 0.373) | 0.192 |
| Systolic blood pressure, mm Hg - mean (SD) | 117.2 (9.8) | -0.046 (-0.142 to 0.050) | 0.341 |
| Diastolic blood pressure, mm Hg - mean (SD) | 73.7 (6.4) | -0.030 (-0.177 to 0.117) | 0.685 |
| Heart rate, bpm - mean (SD) | 75.0 (12.6) | 0.068 (-0.005 to 0.141) | **0.068** |
| Temperature (ear), ℃ - mean (SD) | 36.8 (0.3) | 1.033 (-1.661 to 3.727) | 0.448 |
| Blood hemoglobin (Hb), g/dL - mean (SD) | 13.2 (1.1) | -0.540 (-1.358 to 0.277) | 0.192 |
| Blood thrombocytes, 10^9^/L - mean (SD) | 265.4 (56.1) | 0.003 (-0.014 to 0.020) | 0.708 |
| Plasma alanine aminotransferase (ALAT), U/L - mean (SD) | 19.1 (17.5) | -0.045 (-0.098 to 0.008) | **0.095** |
| Plasma D-dimer, mg/L - mean (SD) | 0.19 (0.13) | -0.160 (-7.604 to 7.283) | 0.966 |
| Plasma ferritin, μg/L - mean (SD) | 85.1 (59.7) | -0.011 (-0.027 to 0.004) | 0.150 |
| Estimated glomerular filtration rate (eGFR) bedside Schwartz, ml/min/1,73m^2^ - mean (SD) | 104.0 (17.8) | 0.000 (-0.053 to 0.053) | 0.992 |
| Plasma N-terminal prohormone of Brain Natriuretic Peptide (NTproBNP), ng/L - mean (SD) | 45.6 (35.4) | -0.024 (-0.050 to 0.002) | **0.070** |
| Troponin T, ng/L - mean (SD) | 3.8 (2.1) | -0.219 (-0.670 to 0.232) | 0.337 |
| Serum sodium/natrium (Na), mmol/L - mean (SD) | 141.3 (2.0) | -0.294 (-0.762 to 0.173) | 0.214 |
| Serum potassium (K), mmol/L - mean (SD) | 4.0 (0.3) | 0.363 (-2.827 to 3.553) | 0.822 |
| Plasma calcium (Ca), corrected for albumin level, mmol/L - mean (SD) | 2.37 (0.07) | 4.174 (-9.856 to 18.204) | 0.556 |
| Plasma albumin, g/L - mean (SD) | 44.1 (2.5) | 0.052 (-0.330 to 0.435) | 0.786 |
| Thyroid-stimulating hormone (TSH), mU/L - mean (SD) | 2.02 (1.45) | -0.136 (-0.782 to 0.509) | 0.676 |
| Plasma free T4, pmol/L - mean (SD) | 16.3 (2.2) | 0.262 (-0.155 to 0.679) | 0.215 |
| Plasma cortisol, nmol/L - mean (SD) | 388.5 (219.2) | 0.001 (-0.003 to 0.005) | 0.700 |
| Plasma lactate dehydrogenase (LD), U/L - mean (SD) | 160.0 (24.2) | -0.018 (-0.057 to 0.021) | 0.351 |
| Plasma creatine kinase (CK), U/L - mean (SD) | 78.7 (51.2) | -0.001 (-0.020 to 0.018) | 0.887 |
| Plasma total bilirubin, µmol/L - mean (SD) | 10.7 (6.0) | -0.110 (-0.264 to 0.044) | 0.160 |
| Plasma gamma-glutamyl transferase (GT), U/L - mean (SD) | 21.9 (13.4) | -0.036 (-0.107 to 0.034) | 0.306 |
| Plasma prothrombin international normalized ratio (INR) - mean (SD) | 1.04 (0.09) | 0.510 (-10.290 to 11.311) | 0.925 |
| Blood neutrophil granulocytes, 10^9^/L - mean (SD) | 3.4 (1.2) | 0.635 (-0.175 to 1.444) | 0.123 |
| Blood lymphocytes, 10^9^/L - mean (SD) | 2.2 (0.6) | -0.117 (-1.637 to 1.403) | 0.878 |
| Blood eosinophil granulocytes, 10^9^/L- mean (SD) | 0.1 (0.1) | -4.520 (-14.571 to 5.532) | 0.374 |
| Blood monocytes, 10^9^/L - mean (SD) | 0.5 (0.2) | 0.720 (-5.557 to 6.998) | 0.820 |
| Blood basophil granulocytes, 10^9^/L - mean (SD) | 0.0 (0.02) | -40.604 (-85.741 to 4.532) | **0.077** |
| Blood glycated hemoglobin (HbA1c), mmol/mol - mean (SD) | 33.9 (3.4) | 0.008 (-0.266 to 0.282) | 0.954 |
| Serum glucose, mmol/L - mean (SD) | 5.1 (0.7) | 1.103 (-0.137 to 2.343) | **0.081** |
| Plasma Vitamin B9, folic acid, nmol/L - mean (SD) | 13.1 (9.6) | 0.007 (-0.092 to 0.106) | 0.891 |
| Plasma Vitamin D, nmol/L - mean (SD) | 56.4 (25.9) | 0.004 (-0.033 to 0.040) | 0.845 |
| Plasma vitamin B12, cobalamin, pmol/L - mean (SD) | 431.6 (171.9) | 0.002 (-0.004 to 0.007) | 0.557 |
| Venous pH - mean (SD) | 7.37 (0.03) | 10.046 (-22.757 to 42.850) | 0.544 |
| Venous HCO3, bicarbonate, mmol/L - mean (SD) | 25.8 (1.9) | -0.056 (-0.560 to 0.447) | 0.824 |
| Venous partial pressure CO_2_ (pCO_2_), carbon dioxide, kPa - mean (SD) | 6.2 (0.7) | -0.534 (-1.882 to 0.814) | 0.433 |
| Serum glial fibrillary acidic protein (GFAP), pg/m - median (IQR) | 56.8 (30.8) | -0.010 (-0.029 to 0.008) | 0.263 |
| Serum neurofilament light chain (NfL), pg/mL - mean (SD) | 4.6 (1.7) | -0.034 (-0.591 to 0.523) | 0.903 |
| Ratio forced expiratory volume after 1 second and forced vital capacity (FEV1/FVC) ^e^ - mean (SD) | 0.86 (0.06) | 13.638 (-2.768 to 30.045) | 0.102 |
| Percentage of predicted FVC ^e^ - mean (SD) | 99.5 (10.0) | -0.012 (-0.112 to 0.088) | 0.817 |
|  |  |  |  |
|  |  | CFQ 12 months | |
| Immunological markers |  | B (CI) | p-value |
| Leukocytes, 10^9^/L - mean (SD) | 6.2 (1.5) | 0.364 (-0.262 to 0.989) | 0.251 |
| Plasma SARS-CoV-2 * total antibody titer (IgM+IgG) - median (IQR) | 2.5 (14.3) | 0.001 (-0.033 to 0.035) | 0.935 |
| TCC */C5b-9, CAU*/mL - median (IQR) | 0.18 (0.17) | 0.520 (-1.307 to 2.347) | 0.573 |
| MCP-1 */CCL2, pg/mL - mean (SD) | 12.20 (8.58) | 0.074 (-0.083 to 0.230) | 0.350 |
| IP10 *, pg/mL - median (IQR) | 139.6 (82.1) | -0.002 (-0.017 to 0.012) | 0.751 |
| RANTES */CCL5, pg/mL - median (IQR) | 272.1 (416.6) | -4.279E-5 (-0.001 to 0.000) | 0.855 |
| Growth/Differentiation factor 15 (GDF-15), ng/mL - median (IQR) | 0.37 (0.12) | 0.455 (-3.466 to 4.376) | 0.818 |
| High-sensitivity assay of C-reactive Protein (hsCRP), mg/L - median (IQR) | 1.0 (2.2) | -0.005 (-0.299 to 0.289) | 0.972 |
| IgG-type antibody against the RBD of SARS-CoV-2, BAU*/mL - median (IQR) | 1005.6 (1621.5) | 0.000 (0.000 to 0.001) | **0.074** |
| Plasma Immunoglobulin A (IgA), g/L - mean (SD) | 1.6 (0.8) | -0.983 (-2.210 to 0.244) | 0.115 |
| Plasma Immunoglobulin G (IgG), g/L - mean (SD) | 11.1 (2.4) | 0.538 (0.154 to 0.922) | **0.007** |
| Plasma Immunoglobulin M (IgM), g/L - mean (SD) | 1.4 (0.5) | 0.597 (-1.310 to 2.504) | 0.535 |
| Serum IgG+IgM against the SARS-CoV-2 Nucleocapsid antigen - median (IQR) | 26.6 (74.7) | -0.004 (-0.018 to 0.010) | 0.562 |
| Plasma Complement C3 (C3bc), ng/mL - mean (SD) | 3.7 (1.4) | 0.524 (-0.143 to 1.190) | 0.122 |
| Plasma Tumor Necrosis Factor (TNF), pg/mL - median (IQR) | 6.4 (8.3) | -0.015 (-0.177 to 0.147) | 0.852 |
| Plasma Interferon Gamma (IFN-γ), pg/mL - median (IQR) | 1.1 (1.5) | 0.231 (0.029 to 0.433) | **0.026** |
| Plasma IL-1β *, pg/mL - median (IQR) | 0.63 (0.97) | 0.662 (-0.514 to 1.838) | 0.266 |
| Plasma IL-2, pg/mL | 0.8 (2.3) | 0.456 (-0.181 to 1.093) | 0.159 |
| Plasma IL-4, pg/mL | 1.4 (0.6) | 0.769 (-0.799 to 2.336) | 0.332 |
| Plasma IL-7, pg/mL | 11.5 (12.3) | 0.025 (-0.047 to 0.096) | 0.496 |
| Plasma IL-8, pg/mL | 0.2 (1.9) | 0.299 (-0.141 to 0.739) | 0.180 |
| Plasma IL-9, pg/mL | 74.8 (137.9) | -0.003 (-0.009 to 0.004) | 0.380 |
| Plasma IL-12p70, pg/mL | 1.4 (4.0) | 0.217 (-0.053 to 0.488) | 0.114 |
| Plasma IL-13, pg/mL | 0.3 (0.6) | -0.134 (-0.998 to 0.730) | 0.759 |
| Plasma IL-17a, pg/mL | 2.1 (2.33) | 0.087 (-0.474 to 0.649) | 0.758 |
| Plasma Eotaxin-1/CCL11, pg/mL | 14.0 (7.4) | -0.012 (-0.185 to 0.161) | 0.889 |
| Plasma MIP-1α */CCL3, pg/mL | 0.7 (0.4) | 0.357 (-2.244 to 2.958) | 0.786 |
| Plasma MIP-1β/CCL4, pg/mL | 26.0 (39.2) | -0.010 (-0.032 to 0.012) | 0.355 |
| Plasma GM-CSF *, pg/mL | 0.1 (0.6) | 0.297 (-0.338 to 0.933) | 0.355 |
| Plasma basic fibroblast growth factor (BasicFGF), pg/mL | 4.0 (5.9) | 0.074 (-0.070 to 0.218) | 0.309 |
| Epstein-Barr Virus (EBV) infection status ^f^ |  | -0.896 (-3.077 to 1.285) | 0.417 |
| ... No prior infection - number (%) | 22 (23.9%) |  |  |
| ... Prior infection - number (%) | 70 (76.1%) |  |  |
|  |  |  |  |
|  |  | CFQ 12 months | |
|  |  |  |  |
| Autonomic markers |  | B (CI) | p-value |
| pNN50 *, % - median (IQR) | 38.6 (37.8) | -0.042 (-0.084 to 0.000) | **0.049** |
| VLF_abs_ *, ms^2^ - median (IQR) | 604.0 (780.5) | 0.000 (-0.001 to 0.000) | **0.014** |
| ln-transformed LF_abs_ *, ms^2^ - mean (SD) | 6.3 (1.0) | -0.900 (-1.805 to 0.006) | **0.051** |
| ln-transformed HF_abs_ *, ms^2^ - mean (SD) | 6.6 (1.2) | -0.609 (-1.366 to 0.148) | 0.113 |
| ln-transformed LF/HF - median (IQR) | -0.1 (1.24) | -0.013 (-0.985 to 0.959) | 0.979 |
| r-MSSD *, ms - mean (SD) | 74.4 (32.8) | -0.029 (-0.051 to -0.007) | **0.011** |
| SDNN *, ms - mean (SD) | 70.4 (40.7) | -0.040 (-0.068 to -0.013) | **0.004** |
| RR-interval *, ms - mean (SD) | 854.1 (111.7) | -0.013 (-0.021 to -0.005) | **0.002** |
| Total Power *, ms^2^ - median (IQR) | 2217.0 (2873.6) | 0.000 (-0.001 to 0.000) | **0.002** |
|  |  |  |  |
|  |  | CFQ 12 months | |
| Cognitive function tests |  | B (CI) | p-value |
| Hopkins Verbal Leartning Test - Revised (HVLT-R) ^g^ Immediate recall - mean (SD) | 25.1 (4.2) | -0.061 (-0.287 to 0.164) | 0.590 |
| HVLT-R Delayed recall - mean (SD) | 9.1 (2.0) | -0.033 (-0.503 to 0.436) | 0.888 |
| HVLT-R Recognition index - median (IQR) | 12.0 (1.0) | -0.201 (-1.353 to 0.951) | 0.729 |
| Digit span ^h^ forwards - mean (SD) | 9.6 (2.2) | 0.028 (-0.417 to 0.473) | 0.901 |
| Digit span backwards - mean (SD) | 5.8 (1.9) | -0.162 (-0.653 to 0.328) | 0.512 |
| Digit span forwards and backwards sum - mean (SD) | 15.4 (3.4) | -0.041 (-0.317 to 0.236) | 0.771 |
|  |  |  |  |
|  |  | CFQ 12 months | |
| Clinical symptoms |  | B (CI) | p-value |
| Chalder Fatigue Questionnaire (CFQ) ^i^ - mean (SD) | 20.0 (4.9) | 0.074 (-0.149 to 0.297) | 0.511 |
| Post-exertional malaise (PEM), items from the DePaul Symptom Questionnaire ^j^ - mean (SD) | 46.4 (28.3) | 0.010 (-0.026 to 0.046) | 0.579 |
| Cognitive symptoms ^k^ - mean (SD) | 8.4 (3.5) | 0.137 (-0.151 to 0.425) | 0.347 |
| Respiratory symptoms ^l^ - mean (SD) | 5.2 (2.0) | -0.112 (-0.589 to 0.365) | 0.641 |
| Autonomic symptoms ^m^ - mean (SD) | 7.7 (3.0) | 0.231 (-0.118 to 0.581) | 0.191 |
| Karolinska Sleep Questionnaire (KSQ) ^n^ - mean (SD) | 39.0 (11.8) | -0.068 (-0.154 to 0.018) | 0.120 |
| Brief Pain Inventory (BPI) ^o^ - mean (SD) | 12.6 (5.8) | 0.014 (-0.159 to 0.188) | 0.869 |
| Headache (symptom 1) ^p^, range 1 to 5 - mean (SD) | 2.9 (1.3) | -0.329 (-1.108 to 0.450) | 0.404 |
| Sore throat/swallowing pain, range 1 to 5 - mean (SD) | 2.2 (1.2) | -0.274 (-1.105 to 0.557) | 0.514 |
| Tender lymph nodes in neck, range 1 to 5 - mean (SD) | 1.4 (1.0) | -0.316 (-1.330 to 0.697) | 0.537 |
| Fever or feeling of fever, range 1 to 5 - mean (SD) | 1.9 (1.0) | -0.600 (-1.548 to 0.348) | 0.212 |
| Shortness of breath, range 1 to 5 - mean (SD) | 2.6 (1.3) | 0.068 (-0.713 to 0.848) | 0.863 |
| Tinnitus, range 1 to 5 - mean (SD) | 2.0 (1.1) | 1.032 (0.158 to 1.906) | **0.021** |
| Cough, range 1 to 5 - mean (SD) | 2.6 (1.2) | -0.407 (-1.223 to 0.409) | 0.325 |
| Runny nose, range 1 to 5 - mean (SD) | 2.9 (1.4) | -0.535 (-1.211 to 0.141) | 0.120 |
| Chest pain, range 1 to 5 - mean (SD) | 1.9 (1.2) | -0.032 (-0.880 to 0.816) | 0.940 |
| Hyperacusis, range 1 to 5 - mean (SD) | 2.1 (1.4) | 0.689 (-0.033 to 1.411) | **0.061** |
| Pain in muscles, range 1 to 5 - mean (SD) | 2.7 (1.5) | 0.078 (-0.610 to 0.766) | 0.822 |
| Nausea, range 1 to 5 - mean (SD) | 2.3 (1.3) | 0.575 (-0.164 to 1.314) | 0.126 |
| Palpitations, range 1 to 5 - mean (SD) | 1.9 (1.2) | 0.320 (-0.538 to 1.177) | 0.461 |
| Dizziness when standing, range 1 to 5 - mean (SD) | 2.7 (1.4) | 0.803 (0.068 to 1.538) | **0.033** |
| Lack of concentration, range 1 to 5 - mean (SD) | 3.3 (1.3) | 0.242 (-0.509 to 0.992) | 0.524 |
| Difficulty in making decisions, range 1 to 5 - mean (SD) | 2.6 (1.3) | 0.334 (-0.428 to 1.096) | 0.386 |
| Difficulty remembering things, range 1 to 5 - mean (SD) | 2.5 (1.3) | 0.407 (-0.388 to 1.203) | 0.312 |
| Fatigue day after exertion, range 1 to 5 - mean (SD) | 3.2 (1.4) | 0.456 (-0.252 to 1.164) | 0.204 |
| Extraordinary fatigue after activity, range 1 to 5 - mean (SD) | 3.3 (1.4) | -0.088 (-0.805 to 0.628) | 0.807 |
| Lack of muscle strength, range 1 to 5 - mean (SD) | 2.8 (1.4) | -0.244 (-0.972 to 0.485) | 0.508 |
| Muscle soreness, range 1 to 5 - mean (SD) | 2.3 (1.3) | 0.864 (0.132 to 1.596) | **0.021** |
| Mental exhaustion, range 1 to 5 - mean (SD) | 2.9 (1.5) | 0.340 (-0.318 to 0.998) | 0.307 |
| Feeling of "drained battery", range 1 to 5 - mean (SD) | 3.0 (1.4) | -0.053 (-0.749 to 0.643) | 0.879 |
| Unrefreshing sleep, range 1 to 5 - mean (SD) | 3.5 (1.3) | -0.199 (-0.967 to 0.569) | 0.607 |
| Numbness in arm or leg, range 1 to 5 - mean (SD) | 2.1 (1.2) | -0.041 (-0.875 to 0.792) | 0.922 |
| Confused or disoriented, range 1 to 5 - mean (SD) | 1.9 (1.2) | 0.685 (-0.140 to 1.511) | 0.103 |
| Pale and cold hands, range 1 to 5 - mean (SD) | 2.4 (1.4) | 0.566 (-0.106 to 1.238) | **0.098** |
| Alternately hot and cold, range 1 to 5 - mean (SD) | 2.6 (1.3) | -0.492 (-1.288 to 0.303) | 0.222 |
| Light sensitivity, range 1 to 5 - mean (SD) | 2.0 (1.3) | 0.523 (-0.230 to 1.276) | 0.171 |
| Multi joint pain, range 1 to 5 - mean (SD) | 2.3 (1.4) | 0.387 (-0.315 to 1.089) | 0.276 |
| Stomach pain, range 1 to 5 - mean (SD) | 2.4 (1.3) | 0.503 (-0.234 to 1.241) | 0.178 |
| Stool changes, loose and/or hard, range 1 to 5 - mean (SD) | 2.3 (1.3) | -0.062 (-0.812 to 0.688) | 0.871 |
| Bloating (symptom 33), range 1 to 5 - mean (SD) | 2.3 (1.2) | 0.218 (-0.605 to 1.041) | 0.599 |
| Positive and Negative Affect Schedule, short-form (PANAS-SF) ^q^ - median (IQR) | 12.0 (8.25) | 0.105 (-0.075 to 0.286) | 0.249 |
|  |  |  |  |
|  |  | CFQ 12 months | |
| Psychological traits/emotions |  | B (CI) | p-value |
| Anxiety subscale, Hospital Anxiety and Depression Scale (HADS) ^r^ - mean (SD) | 8.5 (4.5) | 0.228 (0.010 to 0.446) | **0.040** |
| Depression subscale, HADS ^s^ - mean (SD) | 6.3 (3.9) | 0.188 (-0.059 to 0.435) | 0.134 |
| HADS, sum score - mean (SD) | 14.9 (7.4) | 0.139 (0.007 to 0.272) | **0.039** |
| NEO Five-Factor Inventory-30 (NEO-FFI-30) ^t^ - mean (SD) | 10.9 (6.0) | 0.125 (-0.036 to 0.287) | 0.126 |
| Toronto Alexithymia Scale (TAS-20) ^u^ - mean (SD) | 19.0 (6.9) | 0.039 (-0.102 to 0.181) | 0.581 |
| Penn State Worry Questionnaire (PSWQ) ^v^ - mean (SD) | 52.3 (14.0) | 0.067 (-0.003 to 0.137) | **0.059** |
| General Self-Efficacy Scale, short form (GSE-6) ^w^ - mean (SD) | 16.8 (3.1) | -0.324 (-0.626 to -0.022) | **0.036** |
| UCLA Loneliness Scale ^x^ - mean (SD) | 43.5 (11.9) | 0.066 (-0.014 to 0.146) | 0.107 |
| Brief Illness Perception Questionnaire (BIPQ), modified ^y^ - mean (SD) | 35.6 (12.7) | 0.061 (-0.017 to 0.139) | 0.125 |
| Body Vigilance Scale (BVS) ^z^ - mean (SD) | 14.6 (7.5) | 0.034 (-0.098 to 0.165) | 0.611 |
|  |  |  |  |
|  |  | CFQ 12 months | |
| Function/life events |  | B (CI) | p-value |
| Pediatric Quality of Life (PedsQL) sum/average ^aa^ - mean (SD) | 61.9 (16.0) | -0.067 (-0.129 to -0.005) | **0.034** |
| PedsQL physical ^ab^ - mean (SD) | 56.6 (22.0) | -0.040 (-0.085 to 0.006) | **0.084** |
| PedsQL emotional ^ac^ - mean (SD) | 54.9 (21.9) | -0.039 (-0.083 to 0.005) | **0.083** |
| PedsQL social ^ad^ - mean (SD) | 83.7 (18.3) | -0.063 (-0.115 to -0.011) | **0.018** |
| PedsQL school ^ae^ - mean (SD) | 55.5 (21.0) | -0.012 (-0.059 to 0.035) | 0.613 |
| PedsQL psychosocial ^af^ - mean (SD) | 64.7 (16.5) | -0.057 (-0.116 to 0.002) | **0.060** |
| Physical activity, self-reported, compared to peers - mean (SD) | 5.8 (2.0) | -0.513 (-0.974 to -0.052) | **0.030** |
| Sum score of positive life events last 12 months, Life Event Checklist (LEC) ^ag^ - median (IQR) | 5.0 (6.0) | -0.146 (-0.346 to 0.054) | 0.150 |
| Sum score of all positive life events in past, LEC - mean (SD) | 0.6 (1.4) | -0.807 (-1.466 to -0.148) | **0.017** |
| Sum score of negative life events last 12 months, LEC - median (IQR) | 3.5 (8.0) | 0.154 (-0.019 to 0.327) | **0.080** |
| Sum score of negative life events in past, LEC - median (IQR) | 2.0 (3.0) | 0.031 (-0.316 to 0.379) | 0.858 |
|  |  |  |  |
| NON-BASELINE VARIABLES: |  |  |  |
|  |  | CFQ 12 months |  |
|  |  | B (CI) | p-value |
| Treatment/rehabilitation for post COVID symptoms between 6 and 12 months - number of treated (%) | 57 (63.3%) | -1.722 (-3.603 to 0.159) | **0.072** |
|  |  |  |  |
|  |  | CFQ 12 months | |
|  |  | B (CI) | p-value |
| WHO case definition long covid - 6 months | 69 (74.2%) | 1.133 (-0.997 to 3.264) | 0.293 |
| Fukuda definition PIFS - 6 months | 37 (39.8%) | 1.649 (-0.284 to 3.583) | **0.094** |
|  |  |  |  |
| Comments:  *BMI = Body mass index; ISEI-08 = International Socioeconomic Index 2008; TCC = Terminal complement complex; CAU = Complement arbitrary units; MCP = Monocyte chemotactic protein; IP = Interferon gamma-induced protein; RANTES = Regulated on activation, normal T-cell expressed and secreted; RBD = Receptor binding domain; BAU = Binding antibody units; IL = Interleukin; MIP = Macrophage Inflammatory Protein; GM-CSF = Granulocyte-macrophage colony-stimulating factor; pNN50 = the proportion of successive RRIs with a difference greater than 50 ms; VLFabs = average energy density in the very low frequency band; LFabs = average energy density in the low frequency band (transformed for normal distribution); HFabs = average energy density in the high frequency band (transformed for normal distribution); r-MSSD = the root mean square of successive differences between normal heartbeats (transformed for normal distribution); SDNN = the standard deviation of all RR-intervals (transformed for normal distribution); RR-interval = Mean of all normal RR-intervals during 5-minute resting ECG; Total Power = Sum of energy in VLF, LF, and HF bands. | | | |
| a) Ethnicity was classified as either European (scored as 0) or non-European (scored as 1) based on country of birth of participant and participant's parents.  b) Standardised score calculated according to World Health Organization (WHO) 2006 Child Growth Standards for ages 12-19. For participants above this age, reference values for 19-year-olds were used.  c) The ISEI-08 score of the parent with the highest score. Higher score implies higher socioeconomic status.  d) Having a sibling or parent affected by chronic disease, self-reported.  e) The Global Lung Function Initiative 2012 reference values were used to calculate predicted values.  f) Specific antibody responses were assessed in serum samples using EBV VCA IgM and IgG, and EBV EBNA IgG: Positive IgG antibodies (VCA and/or EBNA) and negative heterophile antibodies at baseline and six months.  g) From the Hopkins Verbal Learning Test revised (HVLT-R). Higher scores imply better recall of words.  h) Test from Wechsler Intelligence Scale for Children, 4th edition (WISC-IV). Higher score implies better short-term memory.  i) 11 items scored on 4-point Likert scales, sum score 0-33, higher score implies more fatigue.  j) From the DePaul Symptom Questionnaire, score 0-100, higher score implies more post-exertional malaise.  k) Cognitive symptoms: Sum score across the three items “memory problems”, “concentration problems” and “decision making problems”, total range is from 3 to 15, where higher scores imply more cognitive symptoms.  l) Respiratory symptoms: Sum score across the two items “dyspnoea” and “coughing”, total range is from 2 to 10, where higher scores imply more respiratory symptoms.  m) Autonomic symptoms: Sum score across the three items “orthostatic dizziness”, “cold and pale hands” and “feeling alternating warm and cold”, total range is from 3 to 15, where higher scores imply more autonomic symptoms.  n) Higher score implies more negative emotions.  o) Higher score implies better sleep.  p) Higher score implies more pain.  q) Symptom scores, 33 different symptoms: 5-point Likert scale, 3 indicates 1-2 times a week.  r) From the anxiety subscale of the Hospital Anxiety and Depression Scale. Higher scores imply more symptoms.  s) From the depression subscale of the Hospital Anxiety and Depression Scale. Higher scores imply more symptoms.  t) Higher scores implies more neuroticism.  u) Higher score implies more difficulty identifying feelings.  v) Higher score implies more worrying.  w) Higher score implies more self-efficacy.  q) Higher score implies more loneliness.  y) Modified to adress illness perception on recent COVID infection. Higher score implies more threatening view of illness.  z) Higher score implies being more attentive to bodily sensations.  aa) Mean of all 23 items in PedsQL. Higher score implies better health-related quality of life.  ab) 8 items. Higher score implies better health-related quality of life.  ac) 5 items. Higher score implies better health-related quality of life.  ad) 5 items. Higher score implies better health-related quality of life.  ae) 5 items. Higher score implies better health-related quality of life.  af) The Psychosocial Health Summary Score is the mean score on the Emotional, Social and School Functioning Scales.  ag) Life Event Checklist (LEC): A total of 48 prespecified life events were presented and the respondents were expected to indicate whether they had encountered the specific event during the last year, and if so, whether they considered the event to be good or bad and assess its subjective impact on a 4-point Likert scale where 0 is “no impact” and 3 is “large impact”. Also, the respondents were allowed to list additional events. Finally, an identical procedure was undertaken for events having occurred any time in the past. Number of positive and negative life events were computed separately for ‘last year’ and ‘any time in the past’; accordingly, sum scores for subjective impact were computed. | | | |

| eTable3. Distribution of ethnic origin and fatigue development (Chalder Fatigue Questionnaire, CFQ sum score) from baseline, six months and 12 months. | | | | | |
| --- | --- | --- | --- | --- | --- |
| Ethnicity | Number (%) | CFQ baseline (mean/SD) | CFQ 6 months (mean/SD) | CFQ 12 months (mean/SD) | Measurements missing - No (%) |
| European | **66 (71.0%)** | **20.2 (4.3)** | **19.7 (4.6)** | **17.5 (4.9)** | **2 ^a^** |
| Non-European/Mixed | **27 (29.0%)** | **19.6 (6.3)** | **20.2 (4.1)** | **22.0 (4.5)** | **1 ^a^** |
| Afghanistan/  Pakistan | 2 (2.2%) | 26.5 (0.7) | 23.5 (5.0) | 23.0 (7.1) | 0 (0) |
| Asia (unspecified) | 1 (1.1%) | 14.0 | 20.0 | 24.0 | 0 (0) |
| China/Vietnam | 1 (1.1%) | 20.0 | 20.0 | 29.0 | 0 (0) |
| India | 1 (1.1%) | 19.0 | 21.0 | 26.0 | 0 (0) |
| Iraq | 1 (1.1%) | 25.0 | 15.0 | 15.0 | 0 (0) |
| Marocco | 1 (1.1%) | 27.0 | 31.0 | 28.0 | 0 (0) |
| Norway/  Philippines | 1 (1.1%) | 19.0 | 22.0 | 26.0 | 0 (0) |
| Norway/  Colombia | 1 (1.1%) | 14.0 | 15.0 | 19.0 | 0 (0) |
| Norway/Ghana | 3 (3.2%) | 24.3 (2.5) | 23.0 (1.7) | 22.0 (6.3) | 0 (0) |
| Norway/  Philippines | 1 (1.1%) | 23.0 | 20.0 | 17.0 | 0 (0) |
| Norway/  South Korea | 1 (1.1%) | 15.0 | 23.0 | 23.0 | 0 (0) |
| Norway/Turkey | 1 (1.1%) | 15.0 | 16.0 | 21.0 | 0 (0) |
| Pakistan | 4 (4.3%) | 14.5 (4.0) | 17.0 (2.2) | 19.3 (4.6) | 0 (0) |
| Philippines | 1 (1.1%) | 3.0 | 15.0 | 18.0 | 0 (0) |
| Somalia | 1 (1.1%) | 26.0 | 25.0 | 27.0 | 0 (0) |
| Sri Lanka | 1 (1.1%) | 19.0 | 22.0 | 22.0 | 0 (0) |
| Sudan | 1 (1.1%) | - | 18.0 | 20.0 | 1 (33.3) ^a^ |
| Chechnya/  Caucasian | 1 (1.1%) | 28.0 | 19.0 | 26.0 | 0 (0) |
| Chechnya | 1 (1.1%) | 25.0 | 25.0 | 26.0 | 0 (0) |
| Turkey | 1 (1.1%) | 13.0 | 16.0 | 19.0 | 0 (0) |
| Vietnam | 1 (1.1%) | 21.0 | 17.0 | 20.0 | 0 (0) |
| All | **93 (100%)** | **20.0 (4.9)** | **19.8 (4.4)** | **18.8 (5.2)** | **3 (1.1%)** |
| 1. All three missing variables were at baseline assessment. | | | | | |

| eTable 4. Comparison of means between reported treatment and no treatment groups. | | | |
| --- | --- | --- | --- |
|  |  |  |  |
| Variable | **Treatment, n = 57** | **No treatment, n = 36** | **Two-sided p-value** |
| Delta CFQ 6 to 12 months ^a^ - mean (SD) | 1.6 (5.0) | -0.1 (4.5) | 0.098 |
|  |  |  |  |
|  | **Specified treatment ^b^, n = 3** | **No treatment, n = 36** | **Two-sided p-value** |
| Delta CFQ 6 to 12 months ^a^ - mean (SD) | 10.3 (3.8) | -0.1 (4.5) | **< 0.001** |
|  |  |  |  |
| Comments:  a) Change in Chalder Fatigue Questionnaire total score from six months to 12 months. Higher score implies more reduction in fatigue  b) Participants who described the type of treatment they had received | | | |
